# Observation of 2D Conduction in Ultrathin Germanium Arsenide Field-Effect Transistors

**DOI:** 10.1021/acsami.0c00348

**Published:** 2020-02-26

**Authors:** Alessandro Grillo, Antonio Di Bartolomeo, Francesca Urban, Maurizio Passacantando, Jose M. Caridad, Jianbo Sun, Luca Camilli

**Affiliations:** † Physics Department “E. R. Caianiello”, University of Salerno, via Giovanni Paolo II n. 132, Fisciano 84084, Italy; ‡ CNR-SPIN Salerno, via Giovanni Paolo II n. 132, Fisciano 84084, Italy; § Department of Physical and Chemical Science, University of L’Aquila and CNR-SPIN L’Aquila, via Vetoio, L’Aquila 67100, Coppito, Italy; ∥ Department of Physics, Technical University of Denmark, Ørsteds Plads, 2800 Kgs. Lyngby, Denmark

**Keywords:** germanium arsenide, 2D conduction, temperature-dependent conduction, field-effect transistors, carrier density, mobility, variable-range hopping

## Abstract

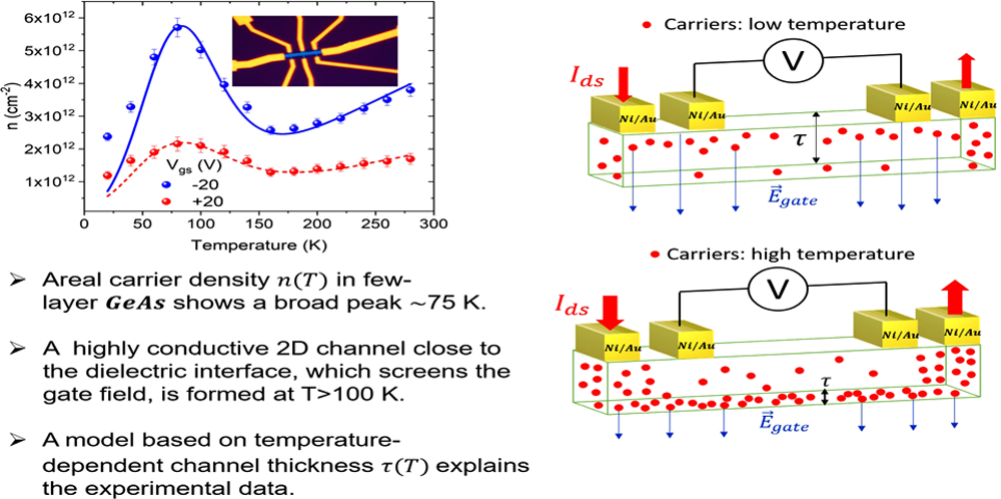

We report the fabrication and electrical characterization of germanium arsenide (GeAs) field-effect transistors with ultrathin channels. The electrical transport is investigated in the 20–280 K temperature range, revealing that the p-type electrical conductivity and the field-effect mobility are growing functions of temperature. An unexpected peak is observed in the temperature dependence of the carrier density per area at ∼75 K. Such a feature is explained considering that the increased carrier concentration at higher temperatures and the vertical band bending combined with the gate field lead to the formation of a two-dimensional (2D) conducting channel, limited to few interfacial GeAs layers, which dominates the channel conductance. The conductivity follows the variable-range hopping model at low temperatures and becomes the band-type at higher temperatures when the 2D channel is formed. The formation of the 2D channel is validated through a numerical simulation that shows excellent agreement with the experimental data.

## Introduction

Since the isolation of graphene,^1^ there has been growing interest in the research of new two-dimensional (2D) materials for the realization of next-generation electronic devices.^2,3^ Several transition metal dichalcogenides (TMDs) have promptly emerged as possible substitutes or complements to graphene^4^ and have already been investigated and proposed for several applications.^5−13^ More recently, the search for new types of 2D materials has led to the investigation of binary compounds of IV and V groups. Indeed, theoretical calculations have indicated that MX compounds, with M = Si, Ge, Sn, or Pb and X = P, As, Sb, or Bi, might have crystalline layered structures with orthorhombic (*Cmc*2_1_ for SiP and *Pbam* for SiP_2_ and GeAs_2_) or monoclinic (*C*2/*m* for GeP, GeAs, and SiAs) symmetries.^14−17^ Several studies have investigated the band gap,^18^ lattice structure,^14^ thermoelectric performances,^19^ and the Hall mobility^20^ of such materials as bulk crystals, but there have been only a few reports on their electrical properties in the form of ultrathin or monolayer flakes.^21,22^

**Figure 1:**
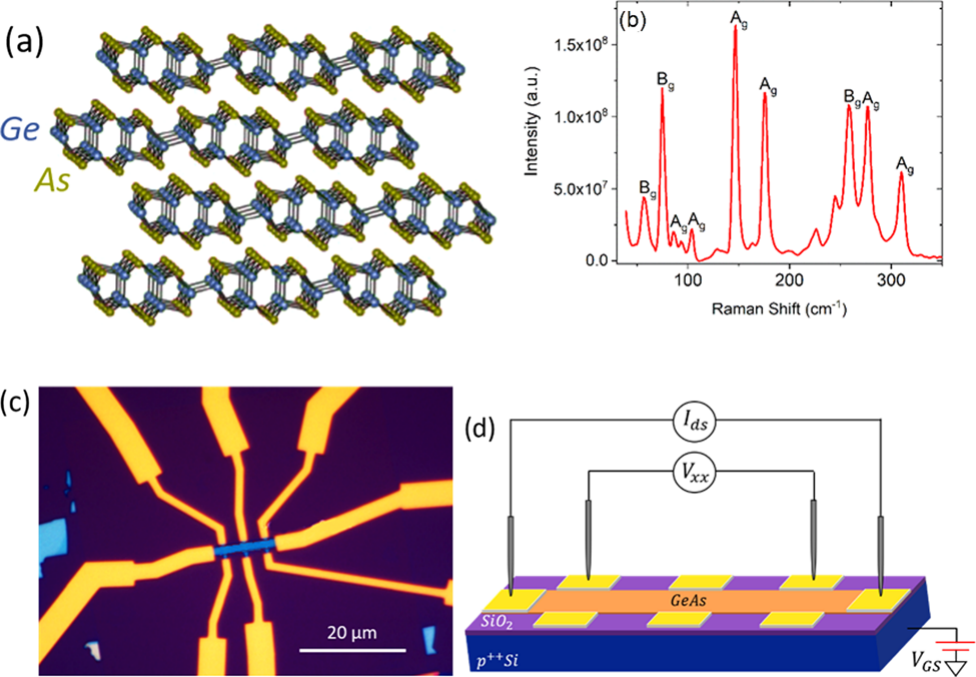
(a) Atom arrangement in GeAs layers. (b) Raman spectrum of a GeAs flake. (c) Optical image of the Hall bar device with Ni/Au electrodes. (d) Four-probe measurement setup.

Among the IV–V compounds, GeAs has attracted attention for possible optoelectronic applications and for its high in-plane anisotropy.^21,22^ GeAs bulk crystalizes in a layered structure, in the space group *C*2/*m*, in which each Ge atom is bonded to one Ge atom and three As atoms forming distorted As_6_@Ge_2_ octahedra.^23^ GeAs layers, terminated by As atoms, are kept together by weak van der Waals forces and show two different types of Ge–Ge bonds. One type is parallel and the other one is perpendicular to the layer plane, highlighting the anisotropic nature of the GeAs crystal structure (see Figure 1a). Similar to TMDs, the GeAs band gap changes according to the number of layers. Numerical calculations and optical band gap measurements indicate that germanium arsenide indirect band gap ranges from ∼0.57 to 0.65 eV for the bulk^19,20^ to 1.6–2.1 eV for the monolayer.^24,25^

In this work, we report the electrical characterization of GeAs back-gate field-effect transistors fabricated using exfoliated flakes of 12 nm thickness. We measure the output and transfer characteristics in a four-probe configuration, highlighting the p-type nature of the GeAs flakes in a vacuum. We investigate the temperature behavior of the transistors in a range from 20 to 280 K, estimating figures of merit such as channel resistivity and field-effect mobility. The free carrier density per area, studied as a function of temperature, shows an unexpected broad peak at around 75 K. To explain this anomaly, we propose a model based on the modulation of the channel thickness with temperature. At low temperatures, the intrinsic carrier concentration in the channel is limited and the electric field of the gate controls the whole thickness of the GeAs flake. At higher temperatures, the increased carrier injection from the contacts and ionization of defects enable the formation of a 2D highly conductive channel close to the dielectric interface, which screens the gate field and confines it to the first few layers of the material. Such a model is corroborated by the estimation of an ∼0.4 nm Debye screening length at room temperature. We compare the numerical simulation of the model with the experimental data obtaining an excellent agreement. To account for the temperature behavior of the resistivity, we use the variable-range hopping (VRH) model at lower temperatures and the thermally activated band-conduction mechanism at higher temperatures. We find that at lower temperatures, the GeAs flake behaves like a three-dimensional system in which the main conduction mechanism is hopping among intragap states, while at higher temperatures, after the formation of the 2D channel, the conduction is dominated by thermal activation of carriers (holes) to the valence band, which leads to the band-type conduction regime.

## Materials and Methods

Ultrathin GeAs flakes were exfoliated from bulk GeAs single crystals using a standard mechanical exfoliation method by adhesive tape. The flakes were transferred onto degenerately doped p-type silicon substrates, covered by 300 nm-thick SiO_2_, on which they were located through optical microscopy. The Raman spectrum of a flake (Figure 1b) was measured under an excitation line of 455 nm by a Thermo Scientific DXR microscope and, in agreement with other studies,^26^ it displays six Raman A_g_ modes at 94, 105, 147, 174, 276, and 308 cm^–1^ and three Raman B_g_ modes at 58, 76, and 257 cm^–1^. By means of an atomic force microscope (AFM), we selected a flake of ∼12 nm thickness, corresponding to about 20 layers, on which we performed standard electron-beam lithography, realizing a typical Hall bar structure, intentionally oriented along one of the crystallographic axes of the material. We then used electron-beam evaporation to deposit 5 nm Ni/40 nm Au as electrodes (Figure 1c). A back-gate contact was formed covering the scratched area of the Si substrate with silver paste.

Electrical measurements were carried out in an Oxford Instrument Teslatron PT cryostat electrically connected with a semiconductor parameter analyzer to perform the standard four-probe characterization of the devices (Figure 1d). All of the measurements were carried out at a pressure of ∼10^–5^ mbar within the temperature range from 20 to 280 K.

## Results and Discussion

**Figure 2:**
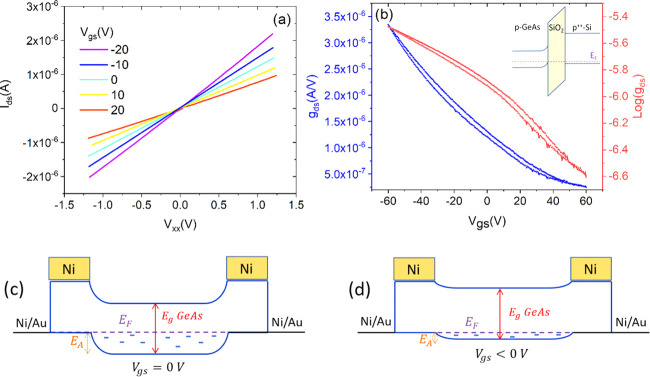
(a) Output characteristic recorded in the four-probe configuration with the *V*_gs_ bias being between −20 and 20 V. (b) Transfer characteristic recorded in the four-probe configuration showed both on linear and logarithmic scales. The inset shows the band diagram in the vertical direction at zero bias clarifying that the device is a normally-on transistor. (c, d) Band diagram along the channel (horizontal) direction, showing alignment between Ni/Au contacts and GeAs at *V*_gs_ = 0 and *V*_gs_ < 0, respectively. The small blue dashes represent the (mostly empty) trap states for holes. The contact region is more doped than the channel (not on scale), thus giving rise to an energy barrier *E*_A_, which is modulated by the gate.

We show in Figure 2 the electrical characterization of the GeAs transistor at room temperature. The output characteristics, i.e., the drain–source current (*I*_ds_) as a function of the voltage drop between two inner contacts (*V*_xx_) with the gate-source voltage (*V*_gs_) as a control parameter, exhibit linear behavior. The application of the gate voltage affects the overall conductance of the sample without modifying the *I*_ds_–*V*_xx_ linearity as expected for four-probe measurements that exclude the effect of the contact resistance.^27−29^ The transfer characteristic, i.e., the *g*_ds_–*V*_gs_ curve (*g*_ds_ = *I*_ds_/*V*_xx_ is the channel conductance) measured over a loop of the gate voltage is reported in Figure 2b. It shows a typical p-type behavior with a modulation that is less than 1 order of magnitude. We did not reach the off-state of the transistor in the applied voltage range, which was limited to avoid the breaking of the device gate dielectric. The p-type behavior is favored by the low work function^15^ of GeAs (∼4 eV) and the higher work function of the degenerate p-Si gate (>5.12 eV) that cause the vertical band alignment shown in the inset of Figure 2b. Furthermore, the Ni Fermi level (work function of 5.15 eV) tends to align below the valence band of GeAs, resulting in ohmic contacts. Furthermore, intrinsic defects such as Ge vacancies, the interaction with the SiO_2_ gate dielectric, and the oxidation of the topmost layers of the flake would act as p-type dopants in the material and provide intragap states.^14,15^ Hole injection from the metal leads makes the area below and near the contacts more doped than the rest of the long channel, thereby giving rise to an energy barrier *E*_A_, which can be modulated by the gate (see Figure 2c,d).^30^

Differently from few-layer TMD-based transistors,^31−35^ the transfer characteristic exhibits negligible hysteresis indicating limited charge trapping during a gate voltage loop.

**Figure 3:**
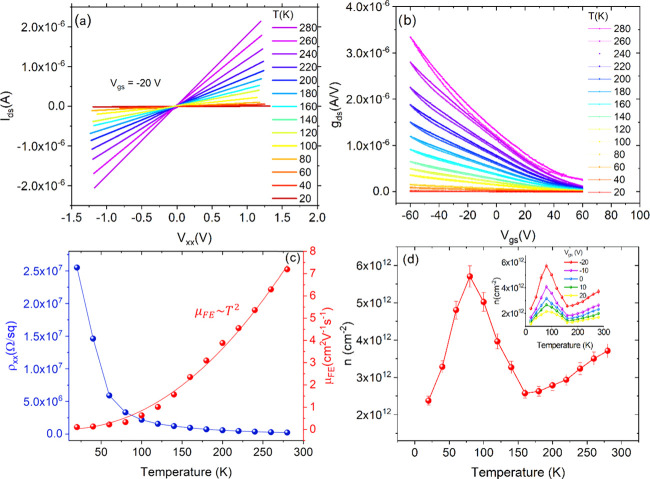
(a) *I*_ds_ versus *V*_xx_ curves at different temperatures. (b) Transfer characteristics recorded for a loop of the voltage bias at different temperatures. (c) Channel resistivity (blue dots) and field-effect mobility (red dots) as a function of temperature. The superposed red line is the quadratic fit. (d) Carrier density *n* versus temperature at *V*_GS_ = −20 V. The inset shows *n* as a function of temperature at several gate biases. The uncertainties are obtained from the standard error propagation.

The temperature dependence of the electric behavior of the device is investigated in Figure 3. We measured the output and transfer characteristics every 20 K in the 20–280 K temperature range (Figure 3a,b). We found a decreasing current and conductance with lowering temperature without any other apparent change in the behavior of the device. From the linear fit of the *I*_ds_–*V*_xx_ curves, recorded at *V*_gs_ = −20 V, we estimated the resistivity ρ of the sample as a function of the temperature (, where *R* is the resistance evaluated from the fit, and *L* = 6 and *W* = 2.7 μm are the channel length and width, respectively). Figure 3c shows a decreasing ρ for increasing temperature, typical of a semiconducting material.

Using the transfer characteristics, considering their nonlinear behavior, we expressed the field-effect transistor (FET) channel conductance as
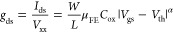
1where μ_FE_ is the field-effect mobility;  is the capacitance per unit area of the gate dielectric, where ϵ_0_ = 8.85 × 10^–14^ F/cm^2^, ϵ_SiO_2__ = 3.9, and *t*_SiO_2__ = 300 nm are the vacuum permittivity, the SiO_2_ relative permittivity, and thickness, respectively; *V*_th_ is the threshold voltage; and α ≥ 1 is a dimensionless parameter that accounts for a possible *V*_gs_—dependence of mobility.^31^ According to eq 1, when the *I*_ds_–*V*_gs_ curve is linear and α = 1, the mobility can be obtained as
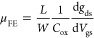
2

To have a fair comparison with the values of ρ obtained from *g*_ds_–*V*_xx_ curves at *V*_gs_ = −20 V, we performed the linear fit of the transfer characteristics in a small interval around *V*_gs_ = −20 V. Figure 3c shows the mobility μ_FE_ as a function of temperature *T*. The quadratic dependence, μ_FE_ ∼ *T*^2^, points toward a mobility dominated by Coulomb scattering due to ionized impurities.^36^ Indeed, it is well known that the mobility is affected by two competitive mechanisms, i.e., the ionized impurity scattering and the phonon scattering.^36^ The first mechanism dominates at lower temperatures and yields increasing mobility with increasing temperature, while phonon scattering becomes the prevailing mechanism at higher temperatures and causes decreasing mobility. As already mentioned, charged impurities leading to Coulomb scattering and increasing μ_FE_ are due to the intrinsic defects of GeAs, the SiO_2_ interface states, and the oxidation of the topmost layers of the flake.

Having computed μ_FE_(*T*) and ρ(*T*) at each of the considered temperatures, we estimated the carrier density per unit area *n* (in cm^–2^) as a function of temperature from the relation
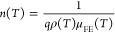
3where *q* is the electron charge. Figure 3d shows that *n*(*T*) exhibits an unexpected and pronounced peak at *T* ∼ 75 K, followed by a smoother increase for *T* > 150 K. In a doped three-dimensional semiconductor, the carrier density would show a different temperature behavior.^36^ Specifically, an initial rapid increase due to the ionization of dopant atoms by thermal energy (freeze-out region, up to ∼150 K for medium-doped Si) would be followed by a plateau over a wide temperature range when complete ionization is reached (extrinsic region, ∼150 to 500 K for medium-doped Si) and would finally evolve in an exponential increase at higher temperatures due to the intrinsic generation of electron–hole pairs (intrinsic region).

**Figure 4:**
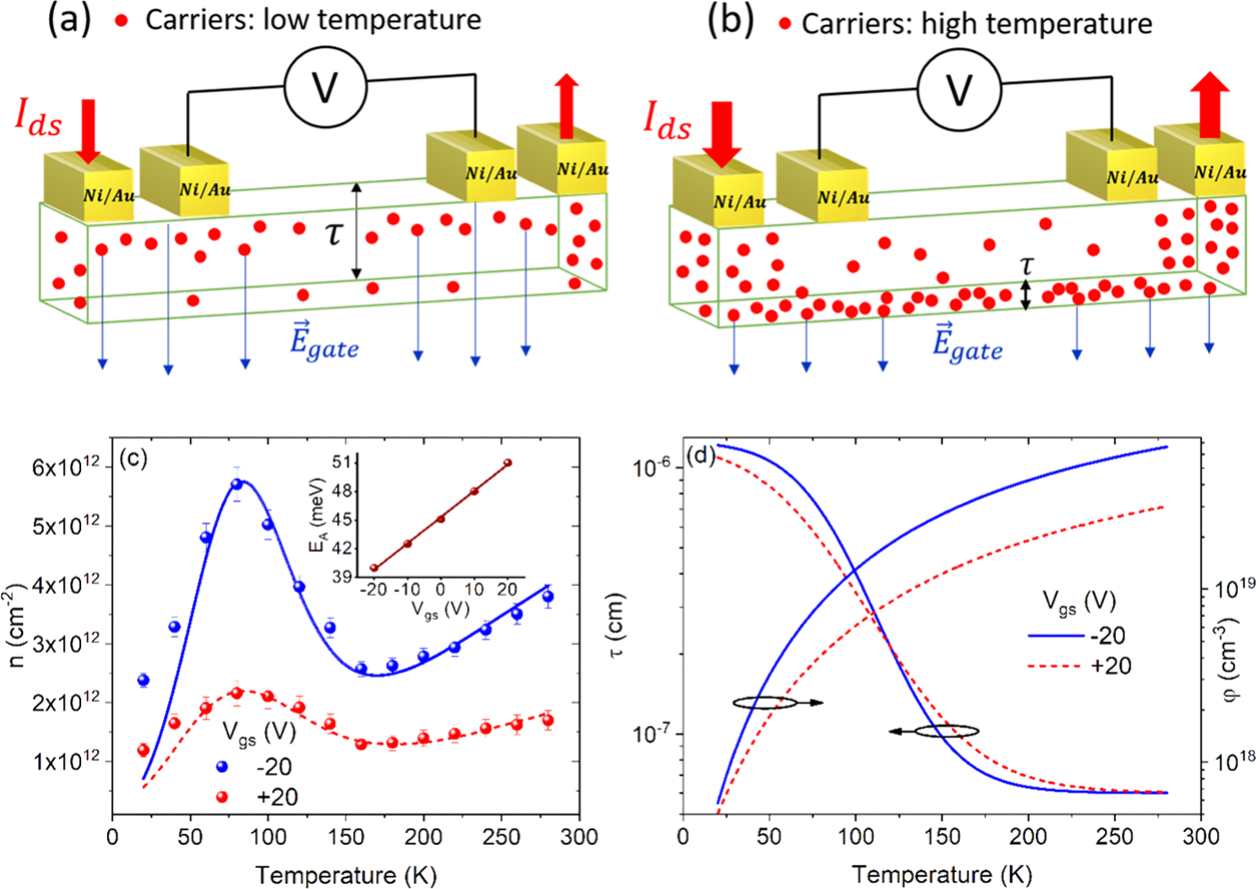
(a) Schematic of the GeAs flake at low temperatures with low carrier density and the electric field of the gate at *V*_GS_ < 0 V (blue arrows) controlling the entire thickness. (b) Schematic of the GeAs flake at high temperatures with the formation of a highly conductive channel confined to a thin interfacial layer, which screens the electric field of the gate (blue arrows). The drawings are not on the scale. (c) Numerical simulation of the 2D carrier density as a function of temperature for *V*_gs_ = ∓20 V (fitting lines superposed the experimental data). The inset shows *E*_a_ as a function of the applied gate potential. (d) Effective thickness of the channel (blue lines) and three-dimensional carrier density (red lines) as a function of temperature for *V*_gs_ = ∓20 V.

To explain the reported anomalous behavior, we propose a model that considers a temperature-dependent thickness of the channel in which most of the conduction occurs. The 2D carrier density per unit area can be expressed as *n*(*T*) = φ(*T*)τ(*T*), where φ is the carrier density per unit volume in cm^–3^ and τ is the thickness of the conducting channel. The current in the GeAs flake is due to the carriers injected from the Ni contacts that overcome the *E*_A_ barrier (Figure 2c,d). Therefore, φ(*T*) in the region between the inner contacts (Figure 4a,b) can be expressed as^37^
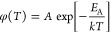
4where *k* is the Boltzmann constant, *E*_A_ is the barrier between contact and channel regions, and *A* is a proportionality constant that can be considered as a fitting parameter of the model. At low temperature (<80 K), there is a limited amount of carriers as well as high intralayer resistance that suppresses the vertical transport with respect to the in-plane one.^30,38^ In this regime, conduction occurs mainly through the layers in closer contact with the metal leads (especially the topmost ones) and the gate electric field controls the entire flake thickness (Figure 4a). With increasing temperature, defect ionization and injection from the contacts increase. The newly available carriers, pushed by the applied gate voltage and the favorable vertical band bending (see the inset of Figure 2b) toward the interface with the gate dielectric, form a 2D channel, which becomes more and more conductive with increasing temperature. This channel now screens the gate field, and any variation of the gate voltage affects mainly the bottommost layers of the flake. In this regime, the conduction is dominated by few atomic GeAs layers closer to the gate, i.e., the effective conducting thickness τ(*T*) controlled by the gate is reduced (Figure 4b).

Such a model is confirmed by the measurement of *n*(*T*) at various gate biases, shown in the inset of Figure 3d. Indeed, the 2D channel at the interfacial region between the gate dielectric and the flake is hampered at positive gate voltages by the opposing gate field. Going to positive gate voltages, the 2D conductive channel is gradually suppressed and so is the carrier density *n* and its peak at ∼75 K.

According to the model, with an appropriate choice of φ(*T*) and τ(*T*), we computed *n*(*T*) and obtained an excellent agreement with the experimental data, as shown in Figure 4c, where we display *n*(*T*) at *V*_gs_ = −20 V (continuous blue line) as well as at *V*_gs_ = 20 V (dashed red line). The slight deviation below 50 K could be caused by additional conduction mechanisms occurring at low temperature (such as hopping, see later). The carrier density per volume φ(*T*), given by eq 4, is shown by the red lines of Figure 4d, with *E*_A_ being obtained as a fitting parameter and displayed as a function of *V*_gs_ in the inset of Figure 4c. For τ(*T*), we used a steplike function, , represented by the blue curves shown in Figure 4d, in which *A* is the initial thickness of the whole flake, i.e., ∼12 nm, *C* is the thickness of a single GeAs layer (0.6 nm),^38^ and *B* is a fitting parameter used to simulate a smooth transition in the 70–150 K range.

The model is further corroborated by the estimation of the Debye screening length , which gives the length over which the electric field strength drops by a factor 1/*e* (here ϵ = 8 is the dielectric constant at room temperature of the material^39^). From our data, we estimate *L*_D_ ∼ 0.4 nm at room temperature, confirming that the gate electric field is substantially screened in the layer closer to the gate.

Consistent with our model, we remark that the formation of a conductive channel near the bottom substrate, screening the gate field, and the presence of an energy barrier caused by the inhomogeneous carrier distribution along the channel thickness have been considered to explain the negative transconductance in thick TMD-based back-gated field-effect transistors.^30^ Furthermore, the appearance of two separate 2D conductive channels, close to the respective gates, due to S-vacancy ionization and gate-field screening, has been recently reported in dual-gated thick MoS_2_ transistors at room temperature. According to such a study, when the temperature is lowered below 80 K, the two separate conductive channels merge into a single one and the top conductive channel governs the transistor behavior.^38^

**Figure 5:**
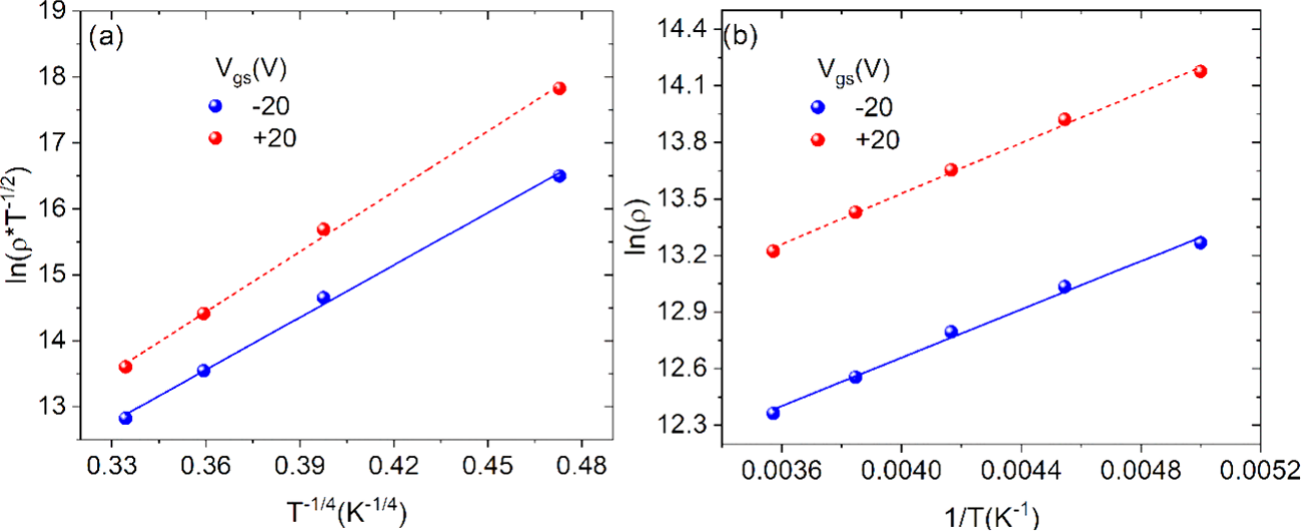
(a) Experimental data and linear fit of ln(ρ*T*^–1/2^) at *V*_gs_ = ∓20 V as a function of *T*^–1/4^ at low temperature, indicating the three-dimensional nature of the investigated system. (b) Experimental data and linear fit of ln(ρ) at *V*_gs_ = ∓20 V as a function of *T*^–1^ at high temperature, indicating the band conduction regime of the investigated system.

We also investigated the resistivity behavior ρ as a function of *T*. The best fitting of the experimental data at low temperature is obtained using the variable-range hopping (VRH) conduction. We notice that VRH is usually observed in amorphous solids as well as in defective systems at low temperature and has been reported in several two-dimensional materials.^40,41^ According to VRH theory, the relation between ρ and *T* can be expressed as^42^
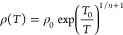
5where ρ_0_ depends on the square root of *T*, *T*_0_ is a constant, and n indicates the dimensionality of the system.^43^Figure 5a shows ln(ρ*T*^–1/2^) as a function of *T*^–1/4^ in the range 20–80 K. The linearity demonstrates that the conduction is bulk type for *T* < 80 K, i.e., it occurs through the entire flake constituting ∼20 atomic layers, as assumed in the proposed model. For *T* > 180 K, ρ shows an exponential increase with the inverse of temperature (Figure 5b). This indicates that the thermal excitation of carriers (holes) to the valence band leads to a band-conduction regime, , as expected after the formation of a highly conductive 2D channel in the GeAs transistor.

## Conclusions

We fabricated back-gate field-effect transistors with ultrathin GeAs films and investigated their electrical properties over a wide temperature range from 20 to 280 K. We found p-type behavior with temperature upon increasing conductivity. We observed that the carrier density in GeAs flakes depends on temperature with a pronounced and broad peak at around 75 K. We proposed a model based on temperature-dependent channel thickness to explain such an anomaly. We showed that the electrical conduction of the GeAs channel can be explained by variable-range hopping at lower temperatures but it becomes the band-type at higher temperatures when a highly conducting 2D channel is formed. The proposed model, validated by numerical simulation, shows an excellent agreement with the experimental data.

This study provides a new understanding of the intrinsic properties of few-layer GeAs as the channel of field-effect transistors, providing evidence of the formation of a 2D channel limited to a single atomic layer and could be applied to other ultrathin layered materials.
